# The Influence of Backpack Weight and Hip Belt Tension on Movement and Loading in the Pelvis and Lower Limbs during Walking

**DOI:** 10.1155/2018/4671956

**Published:** 2018-06-06

**Authors:** Katja Oberhofer, Patrick D. Wettenschwiler, Navrag Singh, Stephen J. Ferguson, Simon Annaheim, Rene M. Rossi, Silvio Lorenzetti

**Affiliations:** ^1^Institute for Biomechanics, D-HEST, ETH Zurich, Leopold-Ruzicka-Weg 4, 8093 Zürich, Switzerland; ^2^Empa, Laboratory for Biomimetic Membranes and Textiles, Lerchenfeldstrasse 5, 9014 St. Gallen, Switzerland; ^3^Swiss Federal Institute of Sport Magglingen (SFISM), Alpenstrasse 16, 2532 Magglingen, Switzerland

## Abstract

The introduction of hip belts to backpacks has caused a shift of loading from the spine to the hips with reported improvements in musculoskeletal comfort. Yet the effects of different hip belt tensions on gait biomechanics remain largely unknown. The goal of this study was to assess the influence of backpack weight and hip belt tension on gait biomechanics. Data from optical motion capture and ground reaction forces (GRF) during walking were acquired in nine healthy male subjects (age 28.0 ± 3.9 years). Six configurations of a commercial backpack were analyzed, that is, 15 kg, 20 kg, and 25 kg loading with 30 N and 120 N hip belt tension. Joint ranges of motion (ROM), peak GRF, and joint moments during gait were analyzed for significant differences by repeated measures of ANOVA with Bonferroni post hoc comparison. Increased loading led to a significant reduction of knee flexion-extension ROM as well as pelvis rotational ROM. No statistically significant effect of hip belt tension magnitudes on gait dynamics was found at any backpack weight, yet there was a trend of increased pelvis ROM in the transverse plane with higher hip belt tension at 25 kg loading. Further research is needed to elucidate the optimum hip belt tension magnitudes for different loading weights to reduce the risks of injury especially with higher loading.

## 1. Introduction

Over the last decade, the use and loading of backpacks have markedly increased for military purposes [1], as well as for school and recreational activities [2, 3]. With increasing loads, reports of musculoskeletal discomfort and injury due to backpack carriage have been growing [3–6]. For instance, a positive correlation was found between backpack weight and dorsal back pain in 3441 students, aged 9 to 15 years [5]; more than 70% of 1122 backpack users, aged 12 to 18 years, were classified as having back pain with significantly poorer general health, limited physical functioning, and other bodily pain [3]; and a significant number of female soldiers reported discomfort in the hip and the foot region following a one-hour march with an average load of 23 kg [4].

Significant research efforts have been directed towards understanding the mechanical predictors of discomfort and risks of injury to improve backpack design and performance, especially associated with heavier loading [7–11]. The measured pressure applied to the shoulder region during backpack carriage has consistently been identified as a clear predictor of discomfort [12–14]. Thereby, it was generally concluded that the hip region is less sensitive to pressure compared to the shoulder region, with suggestions that the static peak pressure in the hip region can be twice as high to cause the same amount of discomfort compared to the shoulder region [14, 15]. Based on these insights from questionnaires and sensor-equipped simulations of upper body biomechanics, hip belts have become state-of-the-art in traditional backpack design to support the shift of heavy loading from the shoulders to the hip, with reports that one-third of the vertical force exerted on the spine during backpack carriage can thus be transferred [16].

Using biomechanical analysis of gait dynamics, higher backpack loading has consistently been found to result in increased vertical and horizontal ground reaction forces (GRF) and an increased hip range of motion (ROM) in the sagittal plane with a tendency of a decreased pelvis ROM in the transverse plane [7–11, 17]. Interestingly, research is indicating that backpack carriage with a hip belt may help in maintaining a more natural pelvis rotation pattern compared to backpack carriage without the hip belt. In particular, Sharpe et al. [18] found a significantly greater ROM of the pelvis in the transverse plane in twelve healthy subjects (age 18–40 years) carrying a backpack with versus without a hip belt while walking on a treadmill at four different speeds, suggesting greater movement stability and a more natural rotation pattern between the pelvis and the thorax compared to backpack carriage without the hip belt. To our knowledge, however, no further research has been conducted to corroborate these findings and extend the analysis of backpack carriage with different backpack weight and hip belt tensions to gait kinematics and kinetics of the pelvis and the lower limbs.

Based on reports in the literature, it is feasible that increased backpack loading is mainly compensated through an adaptation of movement patterns in the upper body, pelvis, and hip, given the fairly consistent results reported for trunk, pelvis, and hip kinematics across the literature despite differences in subject characteristics and loading weight [7–9, 11]. While hip belts are known to result in a load shift off the shoulders to the hip, it remains unclear to which extend the use of hip belts is helping to compensate for the adaptation of movement patterns and load distribution across the trunk, the pelvis, and the lower limbs during dynamic activities such as walking. Thus, the goal of the present study is to analyze the influence of backpack weight and hip belt tension on gait dynamics in healthy adult volunteers in order to guide recommendations for optimal backpack designs to minimize the risk of injury and enhance musculoskeletal performance.

## 2. Materials and Methods

### 2.1. Participants

Nine healthy male subjects participated in this study (age 28.0 years ± 3.9 years, weight 74.1 kg ± 10.5 kg, height 178.8 cm ± 6.0 cm, BMI 23.1 ± 2.5). Only subjects who never had requested any medical advice due to back pain were included. The anthropometrical characteristics of each subject are given in Table 1. All subjects provided written informed consent. The study was approved by the Regional Ethical Committee and was carried out in accordance with “The Code of Ethics of the World Medical Association” (Declaration of Helsinki, amended October 2013).

### 2.2. Backpack Design and Configurations

**Figure 1:**
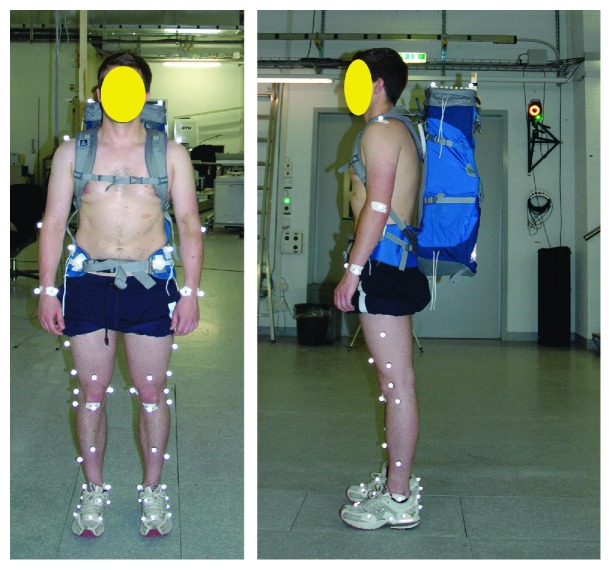
Subject with the Deuter ACT Lite 50+10 backpack with an instrumented hip belt and reflective skin markers for optical motion capture while simultaneously measuring hip belt tension and ground reaction forces.

The commercially available backpack “Deuter ACT Lite 50+10” (Deuter Sport GmbH, Gersthofen, Germany) served as the load carriage system in this study (Figure 1). Its intended use ranges from travelling to trekking and alpine tours. Three different loading weights and two different magnitudes of hip belt tensions were analyzed using the Deuter ACT Lite 50+10, leading to a total of six configurations that were tested on each subject. All subjects wore running shoes and shorts but no shirt during measurements. The height of the backpack was adjusted to the height of each subject according to the manufacturer's recommendations. Here, importantly, the middle of the hip belt was positioned over the hip bones. A custom wooden rack was built inside the backpack that could be adjusted and filled with sand to ensure that the center of mass (CoM) of the backpack was always 30.5 cm above the bottom of the backpack and therefore at the same level and distance to the body center of mass in the midsagittal plane for different weight configurations. The total weights of the backpack were 15.0 kg, 20.0 kg, and 25.0 kg. These weights represent typical weights during hiking or a lightweight in the army. In order to control the hip belt tension, two sensors were used [14] to quantify the force in the belt. The force sensors are using strain gauges and were applied on either the side of the center belt buckle. To reach the hip belt tension of 30 N and 120 N (±1 N), the length of the hip belt was adjusted for each subject during an upright standing position with natural breathing. A detailed description of the custom wooden rack, backpack configuration, and measurement set-up can be found in [14].

### 2.3. Gait Analysis

A 3D motion analysis system with 12 cameras (VICON MX, Oxford Metrics Group, Oxford UK) and five synchronized force plates (Kistler Winterthur, Switzerland, 40x60cm) were used for simultaneously capturing kinematic and kinetic data during level walking with different configurations of backpack carriage. The skin marker configuration was adopted from List et al. [19], except for three markers on the sacrum which could not be placed on the subjects because of backpack carriage (Figure 1). The skin markers were positioned by the same observer for all trials. The joint centers were functionally determined from the trajectories of the skin markers during basic motion tasks according to an established protocol [19]. Following the initial trials of basic motion tasks, each subject conducted eight trials of level walking, including six trials with different backpack configurations and one preassessment and one postassessment of level walking without backpack carriage. The participants were instructed to walk at their own comfortable, self-selected speed. At least five valid double steps were recorded in each trial.

### 2.4. Kinematic and Kinetic Parameters

The following kinematic parameters were calculated from the measured trajectories of the skin markers based on a cluster-based approach of inverse kinematics analysis as described in Schütz et al. [20]: range of motion (ROM) during gait of the ankle, knee, hip and pelvis in the sagittal plane, ROM of the ankle and pelvis in the frontal plane, and ROM of the pelvis in the transverse plane. The following kinetic parameters were derived from the force plate data and normalized to body weight (BW): GRF and time-domain integrals in the cranial-caudal, anterior-posterior, and medial-lateral directions. In addition, the joint moments in the sagittal plane of the knee and the hip during the stance phase of gait were calculated based on the measured trajectories and ground reaction forces using inverse dynamics modelling in a quasistatic approach [21]. All data processing were done using MATLAB (MathWorks, Natick, MA, USA).

### 2.5. Statistical Analysis

One-way ANOVA was performed on all the kinematic and kinetic parameters to identify significant differences in the results with different backpack weights and hip belt tensions as independent variables (Table 1). The significance was set at a *p* value of *p* < 0.05, with Bonferroni tests used for post hoc comparisons. Statistical analysis was conducted using the Statistical Package for Social Sciences for Windows (SPSS Inc., Chicago, IL, USA).

## 3. Results and Discussion

The aim of this study was to investigate the influence of backpack weight and hip belt tension on pelvis and lower limb dynamics during level walking in nine healthy adult male volunteers. The mean values and standard deviations of the kinematic and kinetic outcome parameters during gait for the six different backpack configurations are given in Table 2.

### 3.1. Kinematic Outcome Parameters

**Figure 2:**
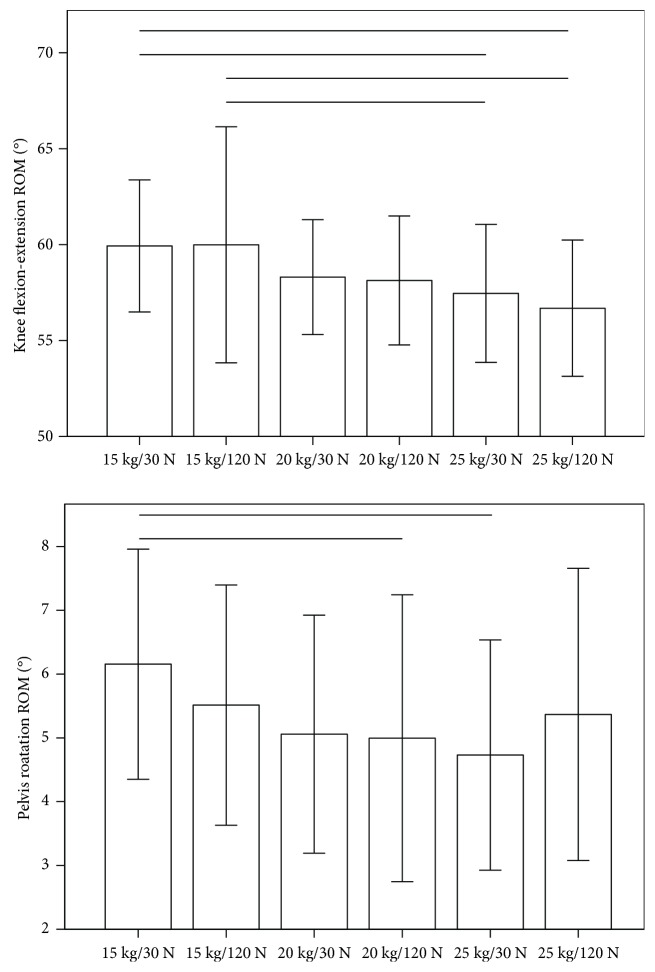
The mean and standard deviations of knee flexion-extension ROM and pelvis rotational ROM during gait for different backpack configurations. The top horizontal bars indicate significant differences between the corresponding trials based on Bonferroni post hoc analysis.

One-way ANOVA revealed a significant effect of different backpack configurations on the knee joint ROM in the sagittal plane (*p* < 0.001), as well as the ROM of the pelvis in the transverse plane (*p* < 0.01) (Table 3). Bonferroni post hoc analysis revealed that the knee joint ROM in the sagittal plane significantly decreased with higher backpack weight from 15 kg to 25 kg for both magnitudes of hip belt tension (Figure 2). Similarly, the pelvis ROM in the transverse plane significantly decreased with increasing backpack weight from 15 kg with a hip belt tension of 30 N to 25 kg with a hip belt tension of 30 N, as well as to 20 kg with a hip belt tension of 120 N (Figure 2). No statistically significant differences in kinematic parameters were found between the two different hip belt tensions at any backpack weight. Interestingly, the pelvis ROM in the transverse plane increased with an increase in hip belt tension from 30 N to 120 N at a constant backpack weight of 25 kg, yet the difference was not statistically significant (Figure 2).

Backpack carriage is known to cause greater inertia and reduced locomotive control on the CoM of the backpack and body system and a reduction in the relative phase of the rotation between the pelvis and the thorax compared to the natural walking pattern without loading [11, 18]. The finding of a significant decrease in the pelvis rotational ROM (i.e., increase in stiffness) with higher backpack loading in the present work is consistent with reported findings [17] and considered a locomotion strategy to facilitate stable forward propagation while minimizing torque generation about the longitudinal body axis that would require large muscular forces to control. A decrease of pelvis rotation in the transverse plane has been associated with an increase in joint moments and power absorption that may potentially be injurious to the hip joint [8, 9]. As such, the present observation of increased pelvis ROM in the transverse plane with higher hip belt tension at 25 kg loading is suggesting that hip belts may support more even joint loading across the lower limbs, pelvis, and upper body which may help to reduce the risks of injury especially with higher loading.

### 3.2. Kinetic Outcome Parameters

**Figure 3:**
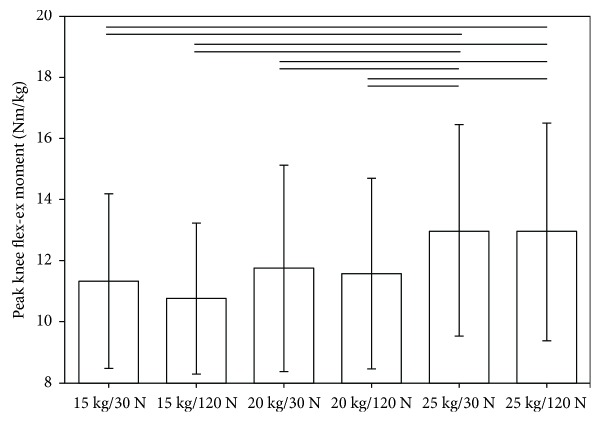
The mean and standard deviations of peak knee joint moment in the sagittal plane during gait for different backpack configurations. The top horizontal bars indicate significant differences between the corresponding trials based on Bonferroni post-hoc analysis.

One-way ANOVA revealed a significant effect of different backpack configurations on the peak magnitudes of the GRF, normalized to BW, in anterior-posterior (*p* < 0.001) and cranial-caudal (*p* < 0.001) directions and the integrals of the normalized GRF over time in anterior-posterior (*p* < 0.001), medial-lateral (*p* < 0.05), and cranial-caudal (*p* < 0.001) directions, as well as the peak knee joint moment (*p* < 0.001) in the sagittal plane (Table 4). Bonferroni post hoc analysis revealed that the kinetic parameters were mainly affected by an increase in backpack weight, and no statistically significant difference was found between the two different hip belt tensions at any backpack weight. Interestingly, the decrease in peak knee flexion-extension moment was not associated with an increase in peak knee flexion-extension ROM (Figure 3), suggesting a redistribution of energy across the lower limbs, the pelvis, and the trunk rather than a local change in knee joint loading.

We did observe neither an increase in peak hip flexion-extension moment nor a significant increase in the hip or pelvis ROM in the sagittal plane with increased loading (Figure 3), which contradicts the findings in the literature in military soldiers as well as school children [7–11]. An increase in the load-induced forward inclination of the trunk and pelvis and hip flexion-extension angle has been considered necessary to counterbalance the hip moments and stabilize the body's CoM [8]. Yet excess forward leaning of the pelvis and trunk while carrying heavy loads for longer duration has also been associated with eccentric contraction of the hamstrings and semispinalis muscles, leading to low back strain, discomfort, and possibly increased the risk of lower back injury [22]. One possible explanation for the different results in the present study compared to other work may be the differences in the backpack design, with the hip belt possibly resulting in an increased stability of the combined CoM of the body and the backpack due to a reduction in the relative motion between the load carriage system and the body. In particular, other authors suggested that the observed increase in pelvis sagittal ROM in military soldiers may be due to poorly designed backpacks with the load not being evenly distributed and/or not snuggly fitted to the upper body [8].

The tighter fitting of the backpack through the hip belt has been suggested to help in slowing and reversing the movement of the backpack prior to the reversal of the trunk and, thus, controlling the rotational torque associated with heavy loading [18]. The relative motion between the load carriage system and the body has been identified as a key predictor of discomfort, especially for the hip region [14]. In particular, Wettenschwiler et al. [14] investigated the musculoskeletal discomfort associated with the relative motion between the backpack and the body during level walking with different loading weight and hip belt tension magnitudes. Interestingly, a higher relative motion of the backpack-body system was found to be associated with higher discomfort in the hip region, while the opposite was found for the shoulder region (i.e., higher relative motion causing less discomfort in the shoulder region). In line with Sharpe et al. [18], it was argued that a stabilization of backpack motion across the hip region with less restriction across the shoulder girdle may enable a more natural motion pattern, thus, resulting in less discomfort. Here, further in-depth analyses of lower and upper body biomechanics during dynamic activities are needed to confirm such benefits for different backpack designs and elucidate the optimum hip belt tension for different loading weights and weight distributions.

### 3.3. Limitations

The effect of hip belts to conventional backpack designs likely depends on a combination of physiological, ergonomic, and biomechanical factors, including the subject's body posture and weight, walking velocity, and magnitude of hip belt tension in relation to loading weight as well as weight distribution with respect to the body CoM. The participants in this study were all of the male sex and young age (28.0 ± 3.9 years, weight 74.1 kg ± 10.5 kg, height 178.8 cm ± 6.0 cm). This group only represents healthy young men such as soldiers. The biomechanics of backpack carriage in similar population groups were previously studied using a range of military load carriage systems with different loading weights and unspecified hip belt tension magnitudes [7, 8, 18, 23]. Our results of the mean knee, hip, and pelvis ROM in the sagittal plane during level walking with 15 kg loading (Table 2) are smaller compared to the reported results by Majumdar et al. [8] in ten healthy male infantry soldiers of similar age and physical constitution walking with a 14.9 kg backpack, that is, a more upright walking pattern with 59.9° (3.4) versus 70.7° (3.3) knee ROM, 39.5° (6.5) versus 47.4° (4.3) hip ROM, and 5.2° (2.1) versus 5.8° (1.9) pelvis ROM. Unlike Majumdar et al. [8], the participants in the present study were free to walk at self-selected, uncontrolled walking speed. Knee, hip, and pelvis ROM in the sagittal plane are known to vary with walking speed, which may have caused some of the differences between the present results and findings in the literature. Yet our results of peak GRF with 25 kg backpack weight are similar to the reported results by Birrell and Haslam [23] in 12 healthy male volunteers (29.2 (9) years) with backpack weight 32 kg, suggesting similar impact forces and musculoskeletal loading.

## 4. Conclusions

The more upright position of the pelvis during gait in our study compared to the literature, despite similar magnitudes of musculoskeletal loading (i.e., backpack weight and measured GRF magnitudes), implies that hip belts may help to stabilize the backpack closer to the body CoM, thus, leading to a change in the kinematics of the multibody dynamic, musculoskeletal system towards a more natural motion pattern. The observed trend of a decrease in peak knee flexion-extension moments with higher hip belt tension for all three loading magnitudes without simultaneous increase in peak knee flexion-extension ROM or peak hip flexion-extension moments further implies that a tighter connection between the backpack and the body via hip belt may help for a more even joint loading across the lower limbs. However, such conclusions need to be treated carefully based on the present data in nine subjects with limited statistically significant support and uncontrolled influence of walking speed on study results. Further research is needed to elucidate the multifactorial influence of backpack design, loading weight, and hip belt tension on musculoskeletal biomechanics in additional subjects, as well as establish upper levels of weight and hip belt tension magnitudes to reduce the risk of injury during dynamic activities.

## Figures and Tables

**Table 1:** Anthropometrical characteristics of participating subjects.

Subject number	#01	#02	#03	#04	#05	#06	#07	#08	#09
Weight (kg)	56.6	74.8	77.2	71.0	95.4	65.4	70.6	70.8	85.2
Height (cm)	180.5	185.0	176.0	175.5	186.0	174.0	174.0	170.0	188.0
Age (years)	23.1	26.6	31.5	25.3	34.3	25.7	27.8	23.5	33.2
BMI (kg/cm^2^)	17.37	21.86	24.92	23.05	27.58	21.60	23.32	24.50	24.11

**Table 2:** Mean range of motion (ROM) (°), peak ground reaction forces (GRF) (%BW), GRF integral (Ns/kg), and peak moment (Nm/kg), as well as the standard deviations (SD) during gait for the six backpack configurations. CC: cranio-caudal; AP: anterior-posterior; ML: medial-lateral; M: moment.

	15 kg/30 N	15 kg/120 N	20 kg/30 N	20 kg/120 N	25 kg/30 N	25 kg/120 N
ROM foot flex-ex	33.1 (5.7)	33.4 (6.0)	32.9 (5.9)	33.2 (5.9)	33.7 (5.8)	35.1 (5.8)
ROM foot inv-ev	4.7 (1.0)	4.3 (0.9)	4.4 (0.8)	4.4 (0.9)	4.3 (1.1)	4.4 (0.9)
^∗^ROM knee flex-ex	59.9 (3.4)	60.0 (6.2)	58.3 (3.0)	58.1 (3.4)	57.5 (3.6)	56.7 (3.6)
ROM hip flex-ex	39.5 (6.3)	41.1 (9.5)	39.5 (7.6)	40.6 (8.3)	40.2 (6.2)	41.6 (8.2)
ROM pelvis tilt	5.2 (2.1)	5.2 (1.6)	5.3 (1.6)	5.2 (1.3)	5.2 (1.3)	5.3 (1.3)
ROM pelvis obli	4.2 (1.3)	4.6 (1.6)	4.3 (1.8)	4.4 (1.8)	4.1 (1.5)	4.5 (1.8)
^∗^ROM pelvis rot	6.2 (1.8)	5.5 (1.9)	5.1 (1.9)	5.0 (2.2)	4.7 (1.8)	5.4 (2.3)
^∗^GRF peak CC	142.6 (10)	143.8 (13.6)	148.2 (13.6)	148.8 (12.4)	155.7 (13.5)	157.1 (14.2)
^∗^GRF int CC	71.7 (3.7)	70.8 (4.5)	75.0 (5.1)	75.2 (4.1)	78.1 (5.1)	79.1 (5.5)
^∗^GRF peak AP	24.2 (4.2)	23.4 (3.5)	24.7 (5.0)	24.6 (4.8)	25.6 (4.9)	26.1 (5.0)
^∗^GRF intdec AP	−3.8 (1.0)	−3.7 (0.9)	−3.9 (1.0)	−3.9 (1.0)	−4.1 (1.0)	−4.1 (1.0)
^∗^GRF intacc AP	4.0 (0.6)	3.8 (0.6)	4.1 (0.8)	4.0 (0.8)	4.2 (0.8)	4.3 (0.8)
GRF peak ML	3.9 (2.3)	4.0 (1.7)	3.5 (1.9)	3.4 (1.9)	3.7 (1.6)	3.5 (2.0)
^∗^GRF integral ML	−2.1 (1.4)	−2.1 (1.6)	−2.3 (1.4)	−2.5 (1.6)	−2.3 (1.5)	−2.7 (1.6)
^∗^M knee peak	11.3 (2.8)	10.8 (2.5)	11.7 (3.4)	11.6 (3.1)	13.0 (3.5)	12.9 (3.6)
M hip peak	13.6 (3.1)	13.1 (3.3)	13.2 (2.5)	13.6 (3.4)	14.2 (3.0)	14.4 (3.2)

^∗^One-way ANOVA revealed a significant effect of independent variables (i.e., backpack weight and hip belt tension) on the resulting parameter.

**Table 3:** The effect of different backpack weights and hip belt tensions on the joint ranges of motion (ROM) during gait. The *F* and *p* values from ANOVA are shown where they are significant (NS: nonsignificant).

Joint	Kinematic parameter	*F* value	*p* value
Ankle	ROM sagittal plane (flexion/extension)	1.088	NS
	ROM transverse plane (in-/eversion)	1.041	NS

Knee	ROM sagittal plane (flexion/extension)	6.239	<0.001

Hip	ROM sagittal plane (flexion/extension)	0.699	NS

Pelvis	ROM sagittal plane (tilt)	0.052	NS
	ROM frontal plane (obliquity)	0.697	NS
	ROM transverse plane (rotation)	3.376	<0.01

**Table 4:** The effect of different backpack weights and hip belt tensions on the ground reaction force (GRF) and peak knee and hip joint moments during gait. The *p* values from ANOVA are shown where they are significant (NS: nonsignificant).

Joint	Kinetic parameter	*F* value	*p* value
GRF	Peak cranial-caudal direction	23.246	<0.001
	Peak anterior-posterior direction	4.569	<0.001
	Peak medial-lateral direction	1.335	NS
	Integral cranial-caudal direction	39.364	<0.001
	Integral deceleration anterior-posterior	3.887	<0.001
	Integral acceleration anterior-posterior	5.708	<0.001
	Integral medial-lateral direction	2.329	<0.05

Knee	Peak moment sagittal plane	5.243	<0.001

Hip	Peak moment sagittal plane	2.153	NS

## Data Availability

The source files from optical motion capture and ground reaction force measurements can be found through the open access repository of the Institute for Biomechanics at http://www.movement.ethz.ch/data-repository.html.
